# Giant cell tumor of the tendon sheath in a 5-year-old child; A case report

**DOI:** 10.1016/j.amsu.2021.102599

**Published:** 2021-07-26

**Authors:** Saywan K. Asaad, Rawa Bapir, Abdulwahid M. Salh, Ari M. Abdullah, Soran H. Tahir, Tomas M. Mikael, Fahmi H. Kakamad, Hunar A. Hassan, Hiwa O. Abdullah, Shvan H. Mohammed

**Affiliations:** a Smart Health Tower, François Mitterrand Street, Sulaimani, Kurdistan, Iraq; b Faculty of Medical Sciences, School of Medicine, University of Sulaimani, Sulaimani, Kurdistan, Iraq; c Department of Urology, Surgical Teaching Hospital, Sulaimani, Kurdistan, Iraq; d Kscien Organization, Hamdi Str., Azadi Mall, Sulaimani, Kurdistan, Iraq; e Sulaimani Teaching Hospital, Sulaimani, Kurdistan, Iraq

**Keywords:** Giant cell tumor, Foot, Tendon sheath, Excision

## Abstract

**Introduction:**

and importance: Giant cell tumors of the tendon sheath in children have rarely been reported in the literature. This study aims to present a case of giant cell tumors of the tendon sheath on the big toe of a 5-year-old child.

**Case presentation:**

A 5-year-old girl presented with a painless swelling over the dorsal aspect of right big toe for 2 weeks. Physical examination revealed non-tender rubbery like swelling over the dorsal aspect of the right big toe. Ultrasound scan of the swelling showed a 17 × 7 mm oval-shaped subcutaneous hypoechoic lesion. Magnetic resonance imaging showed evidence of 20× 8 mm well-defined fusiform soft tissue lesion scalloping the bone. Under general anesthesia, the mass was totally excised. Microscopic sectioning showed a mixture of fibroblasts and histiocyte like cells associated with multinucleated giant cells in the vascular connective tissue stroma with the definite diagnosis of the giant cell tumor of the tendon sheath.

**Discussion:**

These tumors mostly compose of several types of cell like synovial, siderophages, foam, inflammatory and multinucleate giant cells. The major etiological factors that induce development of this tumor could be traumatic, inflammatory, metabolic or neoplastic disease.

**Conclusion:**

although it is a sporadic finding, giant cell tumors of the tendon sheath might affect the lower limb in children. Complete excision is the main modality of treatment.

## Highlights

• A giant cell tumor of tendon sheath is a painless benign soft tissue tumor.
• There are only a few cases that have been reported in the feet of children.
• In this study a case of giant cell tumor of tendon sheath in a 5-year-old child discussed.

## 1 Introduction

Giant cell tumors of tendon sheath (GCTS) are defined as painless benign soft tissue tumors that gradually enlarge in size and mainly contain synovial cells, multinucleated giant cells, siderophages, foam cells and inflammatory cells [1]. This entity was initially described as a fibrous xanthoma by Chassaignac in 1852. However, it has been recently reported by many other names like localized nodular tenosynovitis, pigmented villonodular proliferative synovitis, sclerosing hemangioma, benign synovioma, fibrous histiocytoma of tendon sheath [1,2]. In the clinical view, GCTS appears as a solid painless immobile mass which sometimes forms an extension or satellite lesion linked through a few small fibrous tissue strands [3]. The main factor that influences the color and appearance is, the amounts of hemosiderin, collagen, histiocytes that mix inside the tumor as such, it may vary from grey to yellow-orange with some brownish areas [4]. Individuals at the age of 30–50 are more susceptible to be affected by this condition with the greater chance in women than men. Furthermore, the occurrence of GCTS in children below the age of 10 years is a sporadic entity and even rarer when affects the feet [6]. To our best knowledge, there are only a few cases that have been reported in the feet of children under the age of 10 years [1].

This study aims to present a case of GCTS on big toe in a 5-year-old child with a brief literature review. The report has been arranged in line with SCARE guidelines [7].

**Patient Information:** A 5-year-old girl presented with a painless swelling over the dorsal aspect of right big toe for 2 weeks. There was no history of traumatic events. Past medical, surgical, drug and family history was negative.

**Fig. 1:**
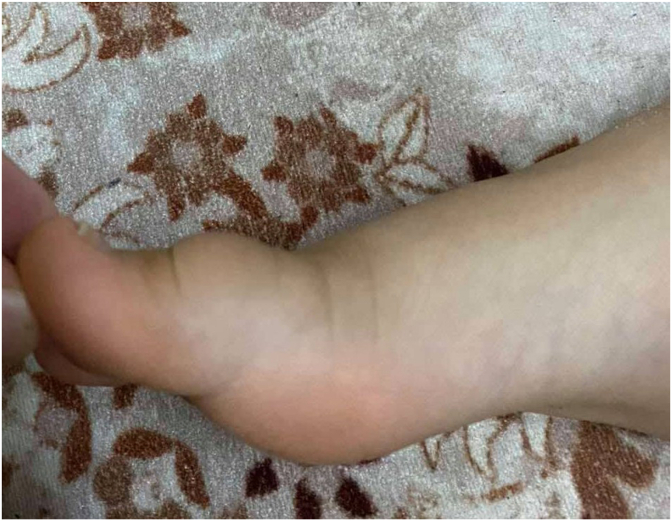
Preoperative photo of the mass (lateral view).

**Clinical Findings:** Physical examination revealed non-tender rubbery like swelling over the dorsal aspect of the right big toe (over the proximal phalanx) with normal overlying skin and normal movements of the metatarsophalangeal and interphalangeal joints (Fig. 1). Vital signs were normal.

**Fig. 2:**
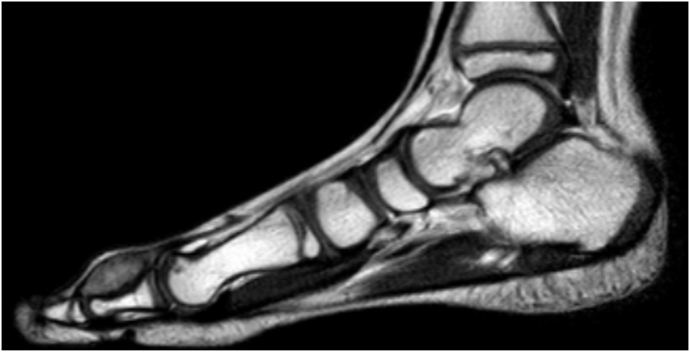
MRI of the foot (Sagital T2WI) shows a well defined fusiform shape soft tissue mass on the dorsum of proximal phalanx of the big toe, with intermediate signal intensity, scalloping underlying bone without destruction.

**Fig. 3:**
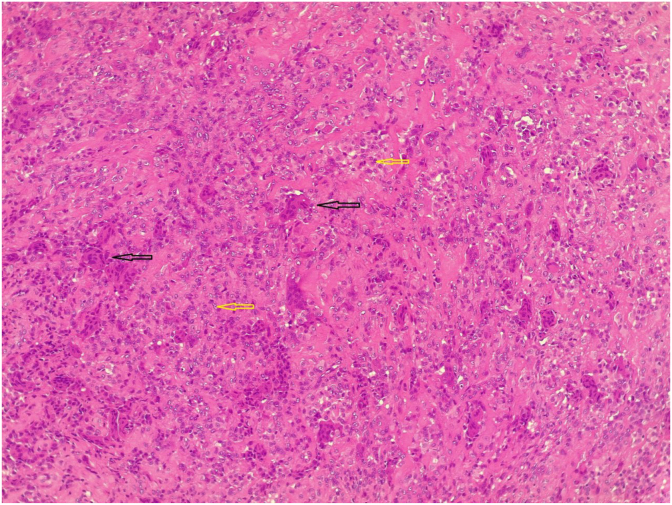
Microscopic picture of the tumor composing of a mixture of osteoclast-like multinucleated giant cells (dark arrows) with bland looking spindle and epithelioid cells (yellow arrows), in a fibrous stroma that contain sidrophages and foamy cells. (For interpretation of the references to color in this figure legend, the reader is referred to the Web version of this article.)

**Diagnostic Assessment:** hematological tests were unremarkable. Ultrasound scan of the swelling showed a 17 × 7 mm oval-shaped subcutaneous hypoechoic lesion at the anterior aspect of the right big toe. Magnetic resonance imaging (MRI) with intravenous gadolinium contrast showed evidence of 20× 8 mm well defined fusiform soft tissue lesion scalloping the bone with no obvious destruction, intermediate signal in T2 with homogenous contrast enhancement (Fig. 2). It was type I according to Al Qattan classification. The overall picture was in favor of soft tissue tumor (see Fig. 3).

**Therapeutic Intervention**: Consent was taken from her parents. The patient was prepared for general anesthesia. In supine position, the mass was totally excised. Histopathological examination of the specimen showed a single rubbery piece of tissue, grey-brown in color, 2.3 × 1.5 × 1.2 cm in size. Microscopic sections showed a mixture of fibroblasts and histiocyte like cells associated with multinucleated giant cells in the vascular connective tissue stroma with the definite diagnosis of the giant cell tumor of the tendon sheath.

**Follow up:** the postoperative period was uneventful. The patient was encouraged to perform all movement of the big toe. She was discharged with oral antibiotics and analgesics. The toe was fully functional.

## 2 Discussion

Giant cell tumor of the flexor tendon sheath was described for the first time in 1852 by Chassaignac [1,2]. It usually arises as a firm, painless immobile mass with different color, including grey, yellow-orange and brownish [5]. This tumor mostly composes of several types of cell like synovial, siderophages, foam, inflammatory and multinucleate giant cells [1]. The major etiological factors that induce development of this tumor could be traumatic, inflammatory, metabolic or neoplastic disease [8]. In the current case, the tumor was grey-brown and contained a mixture of fibroblasts, histiocyte like cells and multinucleated giant cells.

Several studies have claimed that the occurrence of GCTS in individuals under the age of 10 years is very infrequent without existing a reliable explanation [[9], [10], [11]]. In a review by Myers et al. it has been revealed that GCTS occupied 69% in a total of 1301 lesions, but it is worthy to mention that no patient under 10 years old was reported, and only 9 cases of children included in the literature [[12], [13]]. GCTS rarely affects foot (3%) [14]. In addition, Occhipinti et al. reported two cases of GCTS in a 6-year-old female and a 12-year-old male in which both occurred in the toes [15]. It has been found that arising GCTS in the foot and ankle incorporates a wide range of ages between 15 and 59 years [16]. The common site of foot to be affected is the forefoot and hindfoot in the territory of the second toe and it may also arise from a tendon sheath such as the tibialis posterior tendon, flexor digitorum longus tendon, peroneal longus tendon, and small extensor digitorum longus tendon [16]. In the present study, the case was a 5-year-old girl that presented with a painless swelling in the dorsal aspects of her right big toe. There was no history of previous traumatic events as other studies counted trauma as a major etiological factor for the development of this tumor [8].

Ultrasound examination has been suggested to be the first investigation of choice in the diagnosis. It gives information about the vascularity, size, types of the tumor (solid or cystic), the possibility of existing satellite lesions and the relationship of the tumor to the surrounding tissue [[17], [18], [19], [20]]. GCTS usually appears as a homogenous hypoechoic mass on ultrasound [[21], [22], [23], [30]]. Fine needle aspiration cytology (FNAC) may also be helpful in the primary diagnosis of GCTS and aiding in the preoperative planning to prevent local recurrence [[24], [25]]. Radiographs are crucial to reveal cortical erosion of bone or intraosseous involvement [[26], [27]]. Magnetic Resonance Imaging (MRI) is a valuable tool for the diagnosis of soft tissue tumors in foot and ankle [28]. Due to the low signal intensity of GCTS on both T1 and T2 weighted images [29].

Complete resection is the main therapeutic strategy. It has been encouraged to use postoperative adjuvant radiotherapy as well to decrease the chance of recurrence [29].

There are a lot of controversies regarding the factors involving the recurrence of GCTS. These are; hypercellularity and mitotic activity, interphalangeal joint lesion, previous joint diseases and incomplete excision [1,16]. In a review of 29 cases under the age of 18 years, there was no recurrence of GCTS and the authors hypothesized that children might be immuned from recurrence [21]. The recurrence rate of GCTS in the foot and ankle has been reported in adult to be around 20% [16].

In conclusion; although it is a sporadic finding, GCTS might affect the lower limb in children. Complete excision is the main modality of treatment.

## Consent

Written informed consent was obtained from the patient's family for publication of this case report and accompanying images. A copy of the written consent is available for review by the Editor-in-Chief of this journal on request.

## Sources of funding

None is found.

## Provenance and peer review

Not commissioned, externally peer-reviewed.

## Declaration of competing interest

None.
